# Transcriptional profiling of rare acantholytic disorders suggests common mechanisms of pathogenesis

**DOI:** 10.1172/jci.insight.168955

**Published:** 2023-08-22

**Authors:** Quinn R. Roth-Carter, Hope E. Burks, Ziyou Ren, Jennifer L. Koetsier, Lam C. Tsoi, Paul W. Harms, Xianying Xing, Joseph Kirma, Robert M. Harmon, Lisa M. Godsel, Abbey L. Perl, Johann E. Gudjonsson, Kathleen J. Green

**Affiliations:** 1 Department of Pathology, and; 2 Department of Dermatology, Feinberg School of Medicine, Northwestern University, Chicago, Illinois, USA.; 3 Department of Dermatology,; 4 Department of Computational Medicine & Bioinformatics,; 5 Department of Biostatistics, and; 6 Department of Pathology, University of Michigan, Ann Arbor, Michigan, USA.; 7 Robert H. Lurie Comprehensive Cancer Center, Northwestern University, Chicago, Illinois, USA.

**Keywords:** Dermatology, Bioinformatics, Cytoskeleton, Genetic diseases

## Abstract

Darier, Hailey-Hailey, and Grover diseases are rare acantholytic skin diseases. While these diseases have different underlying causes, they share defects in cell-cell adhesion in the epidermis and desmosome organization. To better understand the underlying mechanisms leading to disease in these conditions, we performed RNA-seq on lesional skin samples from patients. The transcriptomic profiles of Darier, Hailey-Hailey, and Grover diseases were found to share a remarkable overlap, which did not extend to other common inflammatory skin diseases. Analysis of enriched pathways showed a shared increase in keratinocyte differentiation, and a decrease in cell adhesion and actin organization pathways in Darier, Hailey-Hailey, and Grover diseases. Direct comparison to atopic dermatitis and psoriasis showed that the downregulation in actin organization pathways was a unique feature in the acantholytic skin diseases. Furthermore, upstream regulator analysis suggested that a decrease in SRF/MRTF activity was responsible for the downregulation of actin organization pathways. Staining for MRTFA in lesional skin samples showed a decrease in nuclear MRTFA in patient skin compared with normal skin. These findings highlight the significant level of similarity in the transcriptome of Darier, Hailey-Hailey, and Grover diseases, and identify decreases in actin organization pathways as a unique signature present in these conditions.

## Graphical abstract

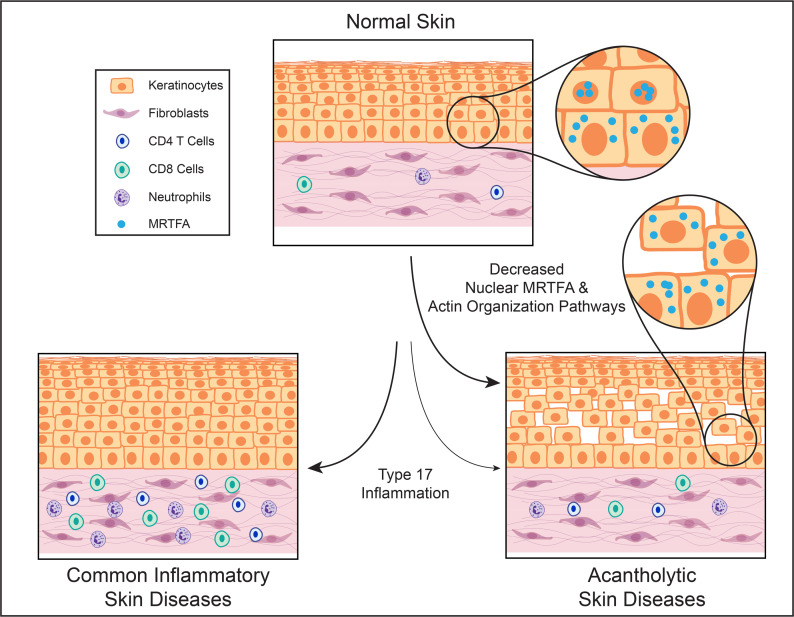

## Introduction

Acantholysis is the loss of adhesion between keratinocytes caused by a disruption in intercellular connections, especially in desmosomes. Acantholysis is a feature shared by several skin diseases, including the nonautoimmune diseases Darier disease (DD), Hailey-Hailey disease (HHD), and Grover disease (GD). DD and HHD are autosomal dominant genodermatoses caused by mutations in the *ATP2A2* or *ATP2C1* genes respectively, while no known mutations have been identified in GD patients (1–3). These genes both encode calcium channels that regulate cytosolic calcium stores, suggesting that dysregulation of calcium homeostasis is a shared feature between DD and HHD. The etiology for GD is unknown, though eruptions are commonly associated with the use of certain cancer therapies (4–7). Whether a disruption in calcium homeostasis occurs in GD, like what is observed in DD and HHD, is unknown. DD, HHD, and GD also share triggers that lead to disease flares, including sweating and exposure to heat. Because the biologic mechanisms of disease are unknown, treatments are currently largely limited to symptom reduction. This lack of mechanistic understanding of these diseases has also limited the development of effective targeted therapies with standard care typically involving the use of topical steroids or retinoids and avoidance of identified flare triggers.

Disruption in desmosome formation has been shown in DD, HHD, and GD and is thought to contribute to the loss of adhesion between keratinocytes in the skin of these patients (8). The mechanisms leading to abnormal desmosome formation and loss of adhesion in acantholytic disease are not well understood. Previous work has identified impaired desmoplakin trafficking in DD patient keratinocytes and SERCA2-deficient cells, the latter of which is a mechanism involving PKCα (9, 10). However, keratinocytes isolated from HHD patients are able to form functioning desmosomes in vitro, suggesting that either assembly of desmosomes is normal and the defect lies elsewhere, or that a second hit is required to reveal a defect (11). Little is known about the cause of desmosome dysfunction in GD, as there have been no mechanistic studies of GD or publications using GD patient–derived keratinocytes. In vitro studies have suggested a role for increased ER stress in mislocalization of cell junction proteins such as E-cadherin and desmosomal components in DD patient keratinocytes (12). Other work has also suggested that a decrease in sphingosine-1-phosphate in DD keratinocytes plays a role in reduced trafficking of cell-cell junction proteins (13). These processes have not yet been demonstrated in HHD or GD, and whether these pathways are important drivers of disease in patients is unknown.

Study of these diseases is further hampered by the lack of animal models that recapitulate human disease symptoms. While mice heterozygous for *Atp2a2* and *Atp2c1* null alleles exist, they do not develop acantholytic skin disorders, but instead develop squamous cell carcinomas as they age (14, 15). As its etiology remains unknown, no animal model exists for GD. To better characterize the shared and divergent molecular and cellular processes driving DD, HHD, and GD we performed transcriptome profiling on lesional skin samples from patients. The results revealed that the gene expression patterns of DD, HHD, and GD are more similar to each other than to the common inflammatory skin conditions atopic dermatitis (AD) and psoriasis (PSO). Pathway analysis revealed unique signatures in DD, HHD, and GD, in particular a downregulation in actin organization pathways, which may highlight novel underlying mechanisms leading to disease in these patients. Further analysis of these actin organization pathways suggested that reduced activity of serum response factor (SRF)/myocardin-related transcription factor A (MRTFA) specifically may be responsible for the decrease in expression of genes associated with regulating actin organization. Validating these findings, staining for MRTFA in the skin of patients with DD, HHD, and GD revealed a decrease in nuclear MRTFA in these patients compared with normal skin.

## Results

### Transcriptome profiling of DD, HHD, and GD patient samples reveals high level of similarity.

**Figure 1:**
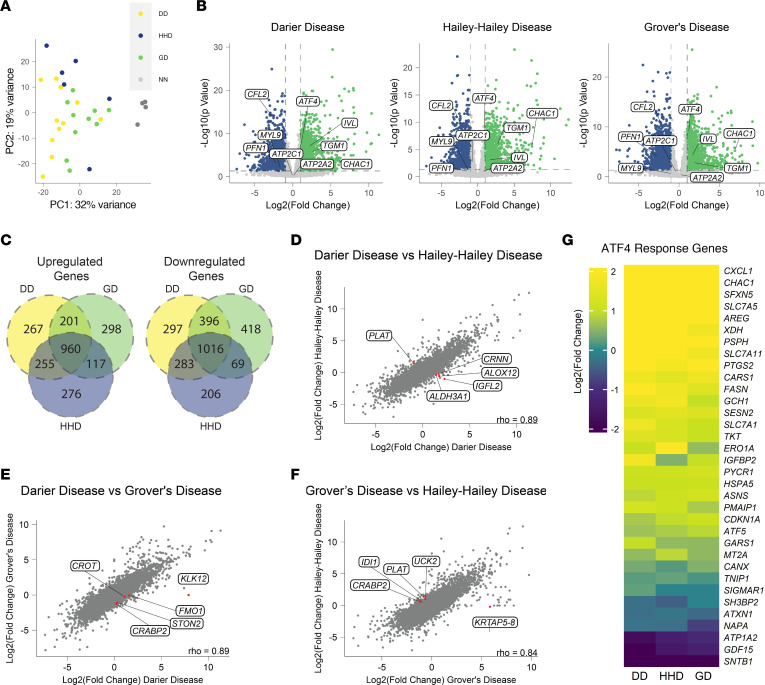
Whole transcriptome profiling of DD, HHD, and GD samples reveals high level of similarity between conditions. (**A**) Principal component analysis of samples from DD, HHD, GD, and normal skin (NN). (**B**) Volcano plots showing significantly upregulated (green) and downregulated (blue) genes in DD, HHD, and GD compared with NN skin. (**C**) Venn diagrams showing overlap in significantly changed genes in DD, HHD, and GD. (**D**–**F**) Correlation analysis of gene expression values from DD, HHD, and GD. Labeled genes are the top 5 ranked genes from Spearman’s correlation, after cutting off genes exhibiting low expression and filtering for genes significantly changed in at least 1 condition. (**G**) Heatmap showing gene expression of ATF4 response genes.

To comprehensively analyze gene expression differences in DD, HHD, and GD patients, we performed RNA-seq on lesional skin samples from 11 DD, 7 HHD, and 10 GD patients (Table 1 and Supplemental Table 1; supplemental material available online with this article; https://doi.org/10.1172/jci.insight.168955DS1). Principle component analysis (PCA) of RNA-seq results from DD, HHD, and GD showed a clustering of disease samples away from normal skin samples from healthy patients (NN); however, the disease samples did not form distinct clusters, but instead formed a large, intermixed cluster (Figure 1A). When compared with normal skin, we observed 1992, 1574, and 1899 significantly downregulated genes in DD, HHD, and GD, respectively, and 1683, 1608, and 1576 upregulated genes in DD, HHD, and GD, respectively (Figure 1B). Previous work has found that mutations in *ATP2A2* or *ATP2C1* often cause a reduction in protein levels of the mutated proteins; however, we did not see changes in gene expression of *ATP2A2* or *ATP2C1* in any conditions (Figure 1B) (16, 17). Comparison of upregulated and downregulated genes showed substantial overlap in significantly changed genes across conditions (Figure 1C). To further determine overlap between samples, Spearman’s correlations were performed on log_2_(fold change) data between each condition, revealing a significant overlap in all pairwise comparisons of each disease (Figure 1, D–F). These observations show that the transcriptional signatures across these diseases share significant similarity and suggest that known changed pathways in one condition would be changed in the others as well. Based on these observations, we tested whether pathways known to be upregulated in DD, such as pathways associated with ER stress, were also present in HHD and GD (12, 18). We performed upstream regulator analysis using Ingenuity Pathway Analysis (IPA) to determine whether transcription factors that regulate ER stress response genes were predicted to be activated and found a significant increase in predicted ATF4 activity compared with control samples, and an upregulation in expression of many ATF4 target genes in DD, HHD, and GD (Figure 1G). These observations suggest that while these diseases do have different etiology and presentation, the underlying changes in the skin transcriptome are similar.

### DD, HHD, and GD share changes in keratinocyte differentiation and cell-cell adhesion pathways.

**Figure 2:**
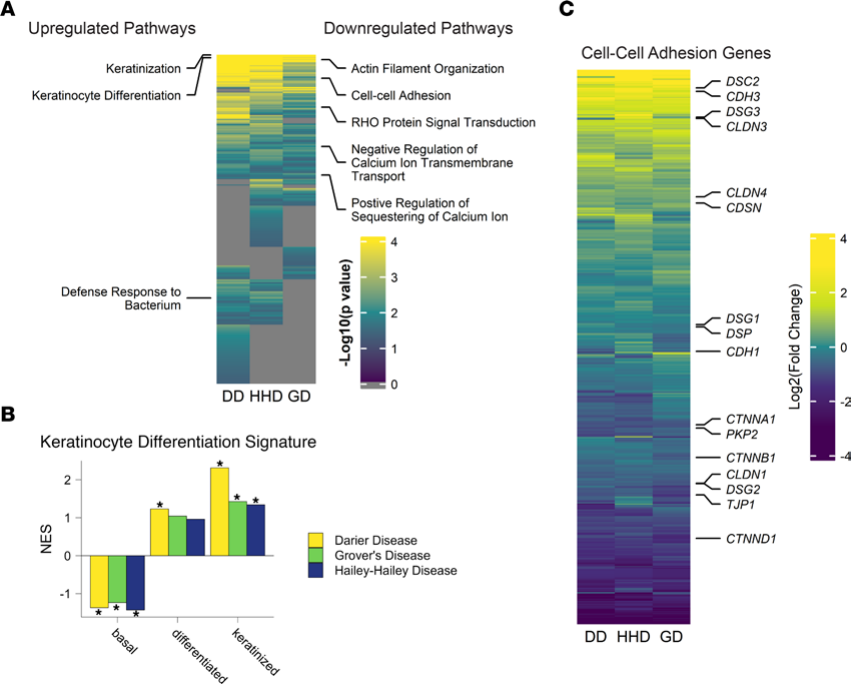
Pathway analysis reveals shared changes in keratinocyte differentiation and cell-cell adhesion in DD, HHD, and GD. (**A**) Heatmap showing GO BP pathways ranked by –log_10_(*P* value) for DD, HHD, and GD. Selected upregulated pathways are noted on the left of the heatmap, while downregulated pathways are noted on the right. (**B**) DD, HHD, and GD gene expression changes were compared to keratinocyte differentiation signatures using GSEA. *FDR < 0.05. NES, normalized enrichment score. (**C**) Heatmap showing log_2_(fold change) values compared with controls for genes annotated to the GO BP cell-cell adhesion pathway.

To identify functional changes in transcriptional profiles from DD, HHD, and GD skin, Gene Set Enrichment Analysis (GSEA) was performed using Gene Ontology (GO) Biological Process (BP) pathways. GSEA identified increases in pathways associated with keratinocyte differentiation and decreases in pathways associated with cell-cell adhesion, actin organization, calcium regulation, and Rho signaling (Figure 2A). To further interrogate the changes in keratinocyte differentiation, we used single-cell RNA-seq data collected from normal skin to create gene set signatures using genes expressed specifically in basal, differentiated, and keratinized keratinocytes (19). Comparison of these gene sets to DD, HHD, and GD using GSEA revealed a decrease in basal associated genes, and a significant increase in keratinized associated genes in all conditions, suggesting a defect in normal keratinocyte differentiation (Figure 2B). We also observed a decrease in pathways associated with cell-cell adhesion, fitting with observations of acantholysis in the skin in these patients (Figure 2C).

### DD, HHD, and GD share greater similarities to each other than to PSO and AD.

**Figure 3:**
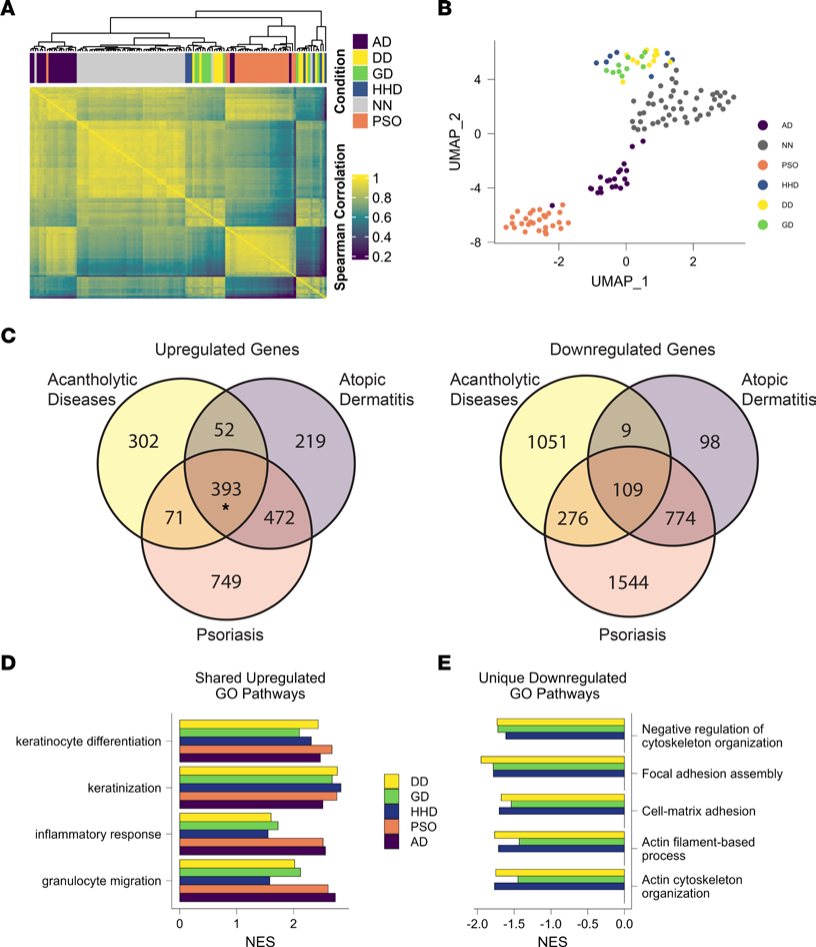
DD, HHD, and GD are more similar to each other than to PSO and AD. (**A**) Heatmap showing Spearman’s correlation values between the batch-adjusted counts from all individual samples grouped using hierarchical clustering. (**B**) Dimensional reduction plot using UMAP on all individual samples. (**C**) Venn diagrams showing overlap in genes upregulated or downregulated in PSO, AD, and combined acantholytic diseases (*P* = 0.4 for downregulated genes and *P < 0.00001 for upregulated genes by permutation test). (**D**) Significantly upregulated GO BP pathways present in all conditions. (**E**) GO BP pathways significantly downregulated in DD, HHD, and GD, but unchanged in PSO and AD.

We next compared DD, HHD, and GD to other more common skin diseases, PSO and AD, to determine shared and unique features across these diseases. To perform direct comparisons between each condition, the DD, HHD, and GD samples were pooled with a publicly available PSO and AD data set, and batch corrected (20). Spearman’s correlation followed by hierarchical clustering showed that PSO, AD, and control samples tended to cluster together, while DD, HHD, and GD formed 2 intermixed clusters (Figure 3A). Similarly, dimensional reduction using uniform manifold approximation and projection (UMAP) showed AD, PSO, and controls separating into distinct clusters, while DD, HHD, and GD cluster into a mixed group (Figure 3B) (21), suggesting that these diseases share greater similarity to each other than to AD or PSO. When comparing overlap in significantly upregulated and downregulated genes between PSO, AD, and combined nonautoimmune acantholytic diseases, samples showed significant overlap in upregulated genes across all diseases, but nonsignificant overlap in downregulated genes between the nonautoimmune acantholytic diseases and PSO and AD, suggesting that the difference in transcriptional signatures is largely driven by downregulated genes in DD, HHD, and GD (Figure 3C). GSEA using GO BP pathways revealed shared overlap in upregulated pathways involved in keratinocyte differentiation and inflammatory responses between DD, HHD, GD, PSO, and AD (Figure 3D). When analyzing downregulated pathways, we observed numerous pathways that were only downregulated in DD, HHD, and GD but not AD and PSO, such as actin cytoskeleton organization and focal adhesion assembly (Figure 3E). While these nonautoimmune acantholytic skin diseases are typically associated with desmosome dysfunction, these observations suggest that there is actin cytoskeleton dysregulation as well (8, 9, 22, 23).

### DD, HHD, and GD share a weak Th17 inflammatory signature that is similar to PSO.

**Figure 4:**
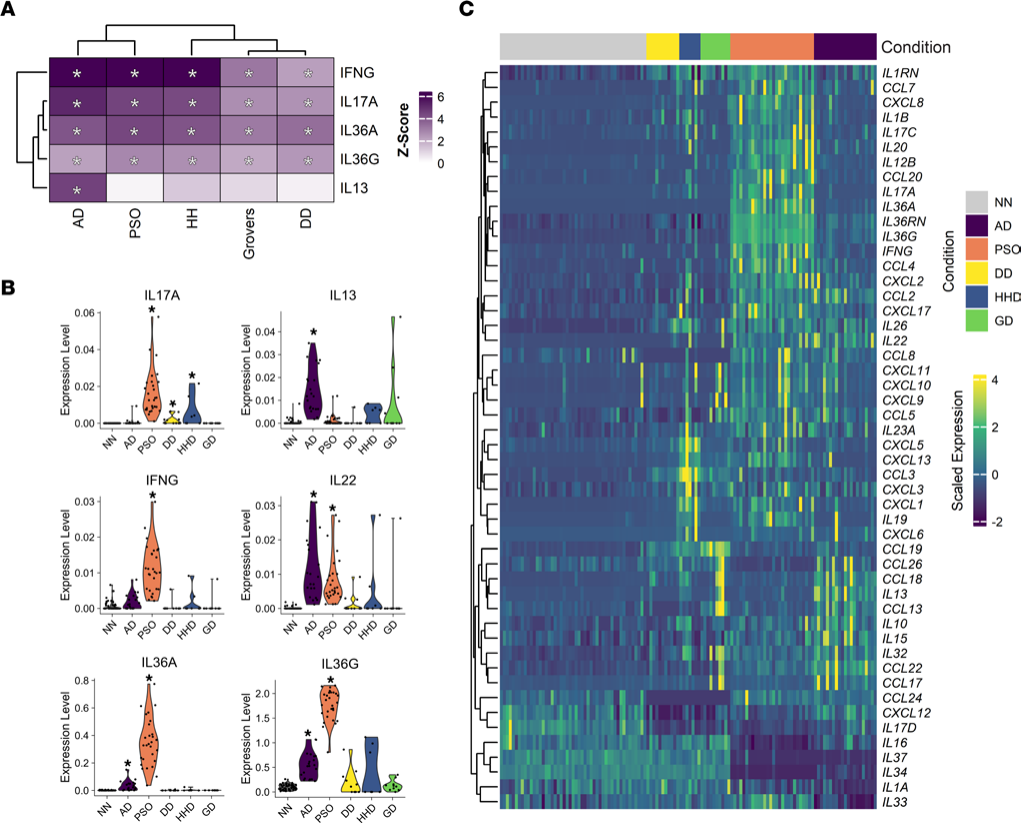
Th17 inflammatory signatures are common across DD, HHD, GD, PSO, and AD, while only AD has Th2 inflammatory signatures. (**A**) Heatmap showing *z* scores of predicted upstream regulators identified using Ingenuity Pathway Analysis (IPA), with a focus on cytokines. (**B**) Violin plots demonstrating expression levels of 6 cytokines from all conditions, including controls (NN). *P < 0.05 by Wilcoxon’s rank-sum test. (**C**) Heatmap depicting scaled expression of cytokines and chemokines across conditions.

Previous work with the AD and PSO data sets has already shown that IL-17 and IFN-γ response pathways are upregulated in both, while IL-13 response pathways are upregulated specifically in AD (20). However, the inflammatory signatures associated with DD, HHD, and GD are unknown; therefore, we used IPA to predict the activity of cytokines using upstream regulator analysis. We observed an increase in predicted IFN-γ, IL-17A, IL-36G, and IL-36A activity in all conditions (Figure 4A). However, the extent of enrichment was lower in DD, HHD, and GD compared with AD and PSO. IL-13 responses were only enriched in AD samples (Figure 4A). Analyzing gene expression of defining cytokines in these response pathways showed modest statistically significant increases in expression of *IL17A* in DD and HHD, while other cytokines were not upregulated in these conditions (Figure 4, B and C). These data suggest that DD, HHD, and GD share a Th17 inflammatory signature, though one that is not as prominent as what is observed in PSO. These observations are in agreement with observations of the immune infiltrate in DD, HHD, and GD, which have described a low level of inflammatory cell recruitment in lesional skin (24).

### DD, HHD, and GD form mixed subgroups with differences in inflammatory signatures.

The observations that DD, HHD, and GD samples form 2 distinct clusters when compared with AD and PSO (Figure 3A) led us to question whether there were distinct subtypes identified in the transcriptomics analysis. To explore this possibility, we split the samples into 2 groups, Group One and Group Two, based on the 2 groups separated in Figure 3A. We did not observe a significant association of any condition to either group, or a significant difference in sex or age between patients in each group (Supplemental Table 2). Spearman’s correlation between log_2_(fold change) data between these 2 groups showed a significant amount of overlap (Supplemental Figure 1A). Interestingly, when we analyzed the top 5 ranked genes from the Spearman’s correlation, we observed that 4 of the 5 genes were typically expressed by inflammatory cells, which suggests there may be an increase in number of inflammatory cells in the skin of patients in Group One. We next performed GSEA using GO BP pathways on each group, and noted significant overlap in pathways enriched in each condition (Supplemental Figure 1B). We observed an increase in skin development pathways and a decrease in pathways associated with actin cytoskeleton in both groups. However, the samples in Group One also had an enrichment in inflammatory pathways not observed in Group Two, suggesting that this group may have an increase in inflammatory signatures (Supplemental Figure 1B). We next performed upstream regulator analysis using IPA, and similarly we observed an increase in predicted activity of inflammatory regulators such as TNF, CSF2, LPS, and NF-κB, which was further enriched in the samples from Group One (Supplemental Figure 1C). Based on these observations of increased inflammatory signatures in Group One, we tested whether there was a predicted increase in percentage of inflammatory cells in the skin using CIBERSORTx to predict cell population proportions. We used single-cell RNA-seq data from normal skin to generate signatures for all cell types in the skin, which was then used to predict cell proportions for those cell types in the bulk RNA-seq samples (25). We observed a significant increase in predicted proportion of neutrophils, CD4^+^ T cells, and CD8^+^ T cells in Group One compared with normal, while there was not an increase in these cell types in Group Two compared to normal and no change in the proportion of other inflammatory cells such as mast cells in either group (Supplemental Figure 1D). These observations suggest that while inflammatory signatures are not dominant in these conditions, there may be a subset of patients with increased inflammation compared with others.

### SRF/MRTF activity is predicted to be downregulated in the skin in DD, HHD, and GD patients.

**Figure 5:**
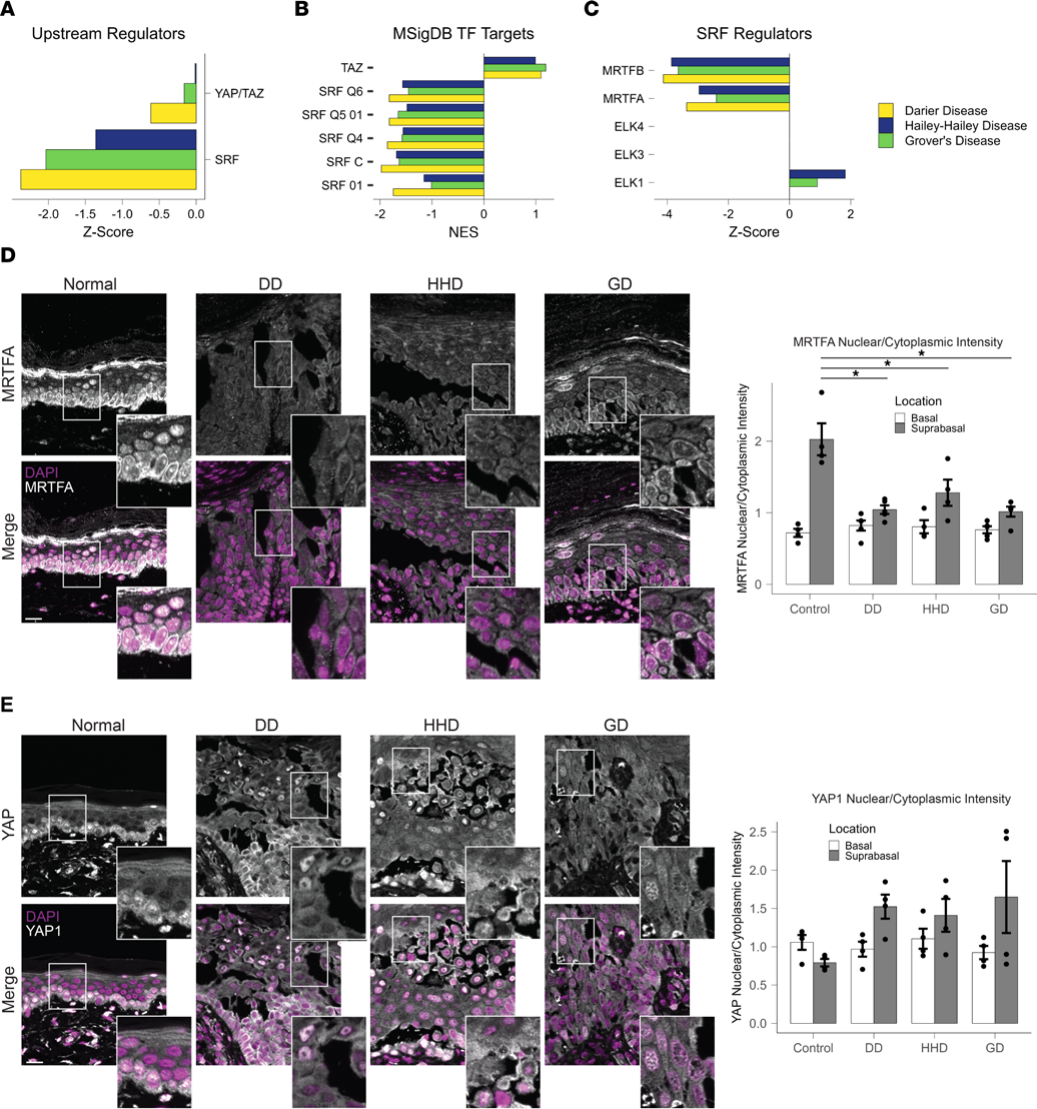
SRF is predicted to be downregulated in DD, HHD, and GD. (**A**) Predicted activity of actin organization–regulating transcription factors identified using upstream regulator analysis. (**B**) Enrichment (NES) of SRF target sequences and TAZ target genes from MSigDB predicted Transcription Factor Targets database. (**C**) IPA upstream regulator analysis showing predicted activity of SRF cofactors. Signatures were not detected for ELK-3 and -4. (**D**) Immunostaining for MRTFA in DD, HHD, GD, and NN skin. Quantification of MRTFA nuclear and cytoplasmic pixel intensities measured in basal and suprabasal cells. Scale bar: 20 μm, *n* = 4 patients per condition. (**E**) Immunostaining for YAP1 in DD, HHD, GD, and NN skin. Quantification of YAP1 nuclear and cytoplasmic pixel intensities measured in basal and suprabasal cells. Scale bars: 20 μm, *n* = 4 patients per condition. *P < 0.05 by 1-way ANOVA with Tukey’s correction for multiple comparisons.

We next sought to examine unique features present in the nonautoimmune acantholytic skin diseases compared with PSO and AD. The downregulation of actin organization pathways was identified as a distinct feature in DD, HHD, and GD compared with AD and PSO (Figure 3E). To explore factors responsible for the downregulation of actin organization pathways in DD, HHD, and GD, we focused on 2 major known transcription factors that regulate genes involved in actin organization: serum response factor (SRF) and yes-associated protein/transcriptional coactivator with PDZ-binding motif (YAP/TAZ). Upstream regulator analysis revealed a predicted decrease in SRF activity, with no significant change in YAP/TAZ in DD, HHD, and GD (Figure 5A), indicating that a reduction in SRF activity may be responsible for the observed downregulation of actin organization pathways in these conditions. Supporting this observation, GSEA of predicted transcription factor target genes using the transcription factor targets database from MSigDB revealed a decrease in genes containing SRF binding motifs, with a trend toward an increase in TAZ target genes (Figure 5B) (26–28). This observation is in line with the upstream regulator findings, again suggesting that SRF activity is reduced in the skin of patients with DD, HHD, and GD. Additionally, we assessed changes in SRF cofactors. The primary mechanisms by which SRF can influence gene expression are through interaction with myocardin-related transcription factors (MRTFs) or interactions with the ternary complex factors (ELK-1, ELK-3, and ELK-4). Upstream regulator analysis predicted decreased activity of MRTFA and MRTFB, while the predicted activity of ELK-1, ELK-3, and ELK-4 remained unchanged in DD, HHD, and GD (Figure 5C). Finally, to validate these observations, we stained patient skin samples for MRTFA and YAP1. We found that the nuclear/cytoplasmic staining intensity for MRTFA was significantly reduced in DD, HHD, and GD samples, while YAP staining, though highly variable, showed a trend toward greater levels (Figure 5, D and E). Given the importance of SRF/MRTFA signaling in epidermal differentiation and barrier formation, its dysregulation in these disorders is likely a contributor to pathogenesis of these diseases (29).

### The decrease in SRF/MRTF is shared between DD, HHD, GD, SAM syndrome, and PF.

We next sought to determine whether the decrease in actin organization–associated pathways and nuclear MRTFA observed in DD, HHD, and GD were specific to these conditions or are shared with other acantholytic skin diseases. To address this we, compared them to the autoimmune acantholytic skin disease pemphigus foliaceus (PF) and the autosomal recessive condition severe dermatitis, multiple allergies, and metabolic wasting (SAM) syndrome. These conditions are both caused by desmosome dysfunction, by autoantibodies that target desmoglein 1 (Dsg1) in PF, or mutations in Dsg1 or desmoplakin in SAM syndrome. We first compared transcriptional signatures from DD, HHD, and GD patients to a previously published RNA-seq data set from PF and SAM syndrome patients (19). For comparison of upregulated and downregulated genes, we combined genes upregulated in at least one condition of DD, HHD, or GD, as there is significant overlap between the conditions, and compared them to PF and SAM syndrome. This comparison showed limited overlap in changed genes (Supplemental Figure 2A). We next analyzed whether there was any overlap in enriched pathways between DD, HHD, GD, PF, and SAM syndrome. GSEA using GO BP pathways identified downregulation of pathways associated with actin organization in PF and SAM syndrome RNA-seq similar to what is observed in DD, HHD, and GD (Supplemental Figure 2B). While there was not a dramatic overlap in individual genes across these conditions, these data suggest that the changes observed in some pathways are preserved across them. Going along with this observation, analyzing predicted upstream regulators using IPA identified a decrease in predicted activity of SRF, MRTFA, and MRTFB in all conditions (Supplemental Figure 2C). These observations suggest that while there may be differences in changed genes between PF, SAM, DD, HHD, and GD, they do share changes in actin organization signatures. To further validate these observations, we stained PF patient biopsies for MRTFA and analyzed changes in nuclear to cytosol ratio in lesional skin. We observed a decrease in MRTFA in lesional skin in PF patient samples, like what was observed in DD, HHD, and GD patient samples (Supplemental Figure 2, D and E).

## Discussion

While DD and HHD are well established to be caused by mutations in the calcium pumps ATP2A2 and ATP2C1, respectively, the mechanism by which these mutations lead to disruption of desmosome function and acantholysis is poorly understood. Even less is known for GD, which has no associated mutational profile and for which no mechanistic studies have been performed to date. Here, we show that the transcriptional profile from lesional skin of GD patients is remarkably similar to DD and HHD patients. Indeed, the level of similarity between these 3 conditions to each other was greater than to other common inflammatory skin diseases AD and PSO. The fact that DD and HHD are caused by mutations in calcium channels raises the question as to whether GD also shares dysregulation of calcium homeostasis, and interestingly we observed changes in pathways associated with calcium regulation in DD, HHD, and GD, though further study would be required to demonstrate calcium dysregulation in GD keratinocytes. The question of calcium dysregulation remains in GD, but this observation suggests that downstream changes are related in these 3 conditions.

When comparing shared pathways between DD, HHD, and GD to AD and PSO we discovered shared upregulation in pathways associated with keratinocyte differentiation. Upregulation of keratinocyte differentiation pathways is commonly observed in chronic skin diseases, including those like AD and PSO (30, 31). This may be caused by the thickening of the epidermis leading to an increase in the proportion of differentiated keratinocytes in the bulk RNA sample. We also observed a modest increase in inflammatory pathways in DD, HHD, and GD, pathways which are more strongly associated with AD and PSO. Given the abundance of inflammatory response targeting therapeutics currently in use for other inflammatory skin conditions, we further investigated the pattern of inflammatory signaling activation in DD, HHD, and GD. The inflammatory signature in DD, HHD, and GD shared greater similarity to PSO than with AD, as characterized by increases in Th17- and Th1-related responses and a lack of Th2-related responses. However, the degree to which these signatures were enriched in DD, HHD, and GD was much lower than what is observed in PSO and AD. Analysis of individual genes showed no significant upregulation of many of the cytokines or chemokines that define these pathways. Interestingly, while doing this analysis we also observed that DD, HHD, and GD patients divided into 2 intermixed groups. As this observation suggested that there may be shared endotypes within these conditions, we further analyzed these 2 groups to understand the differences in transcriptional signatures between them. Our analysis found that while these groups still share the major signatures identified in the original analysis, such as those associated with keratinocyte differentiation and actin organization, we did observe a difference in inflammatory signatures between the groups. There are several possible explanations for this observation. It is possible that there are shared endotypes between patients with DD, HHD, and GD, with some endotypes displaying more severe inflammation compared with others. It is also possible that lesions develop inflammation over time. Indeed, others have shown an increase in transepidermal water loss in lesions from patients with DD and HHD, suggesting barrier defects that can promote inflammation (32). Further study on this topic would be required to determine whether either of these possibilities is correct, or whether there is another explanation for this observation. Interestingly, another report of RNA-seq profiling from 2 patients with HHD demonstrated differences in inflammatory profiles across different patients, with one patient exhibiting a predicted increase in NF-κB compared with another patient, and an increase in NF-κB target cytokines such as IL-8 and CCL20 (33).

Changes in pathways associated with actin organization were specifically changed in DD, HHD, and GD patients, remaining unchanged in PSO or AD compared with normal control patients. The acantholysis observed in DD, HHD, and GD is commonly attributed to desmosome dysfunction. While desmosomes are classically defined as regulators of the intermediate filament cytoskeleton, previous work from our group has demonstrated that desmosomes can regulate actin remodeling as well (34–36). We have also shown that actin is required for trafficking of desmosomal components to form functional desmosomes (35).

The SRF/MRTF transcription factor was predicted to be the upstream regulator driving the observed changes in actin organization pathways. We confirmed the decrease in MRTFA by showing decreased nuclear localization of MRTFA in lesional skin in DD, HHD, and GD patients. Interestingly, epidermal SRF–knockout mice show disrupted formation of desmosomes and an increase in intercellular gaps, suggesting a disruption in cell-cell adhesion similar to the histopathology characterized in DD, HHD, and GD patient skin (29, 37). Previous work from our lab demonstrated that inactivation of SRF/MRTFA results in a decrease in expression of the desmosomal cadherin desmoglein 1 (38). Inhibition of MRTFA has also been shown to increase expression of transcription factors that regulate ER stress, including ATF4, potentially providing a link between these findings and the observations of increased ER stress pathways previously shown in DD and observed in DD, HHD, and GD here (39). Though previous work has shown a role for MRTFA activation in wound healing in a mouse model, we do not yet know whether activation of SRF/MRTFA would reduce disease burden in acantholytic disease (40).

An unanswered question from these studies is whether the changes in actin organization pathways and SRF/MRTF localization are limited to DD, HHD, and GD, or whether they are shared by other skin conditions that present with acantholysis. To address this question, we compared DD, HHD, and GD transcriptomics to other skin diseases that present with acantholysis, PF and SAM syndrome. In these conditions, acantholysis is due to direct disruption of desmosomes, either through the production of autoantibodies against Dsg1 in pemphigus or mutations in Dsg1 or desmoplakin in SAM syndrome. Interestingly, we observed a predicted decrease in SRF/MRTF activity in both PF and SAM syndrome, and a decrease in pathways associated with actin organization. These observations suggest that decreases in these pathways are common across these conditions but does not distinguish between the possibilities that these signatures precede and cause acantholysis, or whether they are caused by acantholysis. Previous work by others has found that activation of RhoA, a canonical upstream activator of MRTF and SRF, can prevent keratinocyte dissociation induced by pemphigus antibodies in vitro (41). Based on these observations, we would propose that dysregulation of SRF/MRTF in DD, HHD, and GD leads to acantholysis in the skin in these patients, though further study will be required to prove this connection and order of events.

## Methods

### Additional gene expression data sets.

The AD and PSO RNA-seq data set was downloaded from the NCBI Gene Expression Omnibus (GEO), accession number GSE121212. This data set was originally described in Tsoi et al. (20). The SAM syndrome data set was downloaded from GEO accession number GSE189096, and was originally described in Godsel et al. (19). The single-cell RNA-seq data set used to generate keratinocyte differentiation genes sets are available in GEO, accession number GSE179162.

### RNA-seq expression profiling of DD, HHD, and GD samples.

RNA was isolated from 10-mm sections of formalin-fixed, paraffin embedded blocks from 11 DD, 7 HHD, 10 GD, and 4 normal samples. RNA was isolated using the E.N.Z.A. FFPE RNA Kit (Omega Bio-tek). Samples were prepared using the Lexogen 3′ QuantSeq mRNA-Seq Library Prep Kit FWD and sequences on the Illumina NovaSeq 6000 System. Quality control and adaptor trimming were performed on sequence reads from the RNA-seq data. STAR alignment (https://github.com/alexdobin/STAR/releases) was used to align the reads to the reference genome (GRCh37), and HTSeq (https://htseq.readthedocs.io/en/latest/install.html#install) was used for gene quantification . To eliminate potential differences caused by sex-specific genes, Y chromosome genes and genes known to be differentially expressed between males and females in skin were removed from further analysis (42). To generate differential expression for DD, HHD, and GD the DESeq2 Bioconductor R package v1.34.0 was used (https://bioconductor.org/packages/release/bioc/html/DESeq2.html).

### Correlation analysis between DD, HHD, GD, PSO, and AD.

Analysis was performed in R v4.1.1 (R Foundation for Statistical Computing). Batch correction was performed using ComBat-seq function under the SVA Bioconductor R package v3.36.0.0 following a published method (43). PCA was computed based on batch-adjusted raw counts using R package pcaMethods v1.86.0. For comparisons between DD, HHD, GD, PSO, and AD, the EdgeR Bioconductor R Package v3.36.0 was used to generate differential expression data from batch-adjusted raw counts and calculate statistically significantly changed genes by ANOVA. Genes that had an FDR-adjusted *P* value of less than 0.05 and |log_2_(fold change)| of greater than 1 in any one condition were used for all further comparison analysis. Spearman’s correlations were calculated using the batch-adjusted raw counts and the *cor* function in R build-in stats package. UMAP plots were created using the Seurat package in R v4.1.1.

### Functional enrichment analysis.

Functional enrichment analysis was performed using the clusterProfiler package in R v4.2.2 and using IPA software (Qiagen). Pathway analysis was performed using GO BP and the Transcription Factor Targets pathways from MSigDB, and were analyzed using GSEA (44).

### Immunofluorescence and image acquisition.

DD, HHD, GD, and normal patient skin samples were fixed in 10% formalin, embedded in paraffin blocks, and cut into 4-mm-thick sections. For immunostaining, paraffin sections were baked at 60°C overnight and deparaffinized with xylene. Samples were then rehydrated through a series of ethanol and PBS washes, and permeabilized in 0.5% Triton X-100 in PBS. Antigen retrieval was performed by incubating the slides in 0.01 M citrate buffer at 95°C for 15 minutes. Samples were blocked in blocking buffer (1% BSA, 2% normal goat serum in PBS) for 1 hour at 37°C. Samples were then incubated in primary antibody overnight at 4°C, followed by washes with PBS and incubation in secondary antibody for 1 hour at 37°C. Images were acquired using an AxioVision Z1 system (Carl Zeiss) with an Apotome slide module, and AxioCam MRm digital camera and a 20× (0.8 NA Plan-Apochromat) objective. Image analysis was performed using ImageJ software (NIH).

### Predicted abundance of inflammatory cells.

Prediction of cell abundance was performed in the bulk RNA-seq data using the CIBERSORTx (45). Briefly, single-cell RNA-seq data from normal skin was used to generate cell-type signatures (single-cell RNA-seq used from GSE162183). Samples were then processed using the CIBERSORTx online tool (https://cibersortx.stanford.edu/).

### Antibodies.

Antibodies used in this study include rabbit anti-YAP1 (14074, Cell Signaling Technology), rabbit anti-MRTFA (PA599446, Thermo Fisher Scientific), and Alexa Fluor 568–conjugated goat anti-rabbit secondary antibodies (Thermo Fisher Scientific).

### Data availability.

RNA-seq data were submitted to NCBI’s GEO repository (GSE233000).

### Statistics.

Permutation analysis for Venn diagrams was performed to test the significance of the overlap. Ten thousand permutations were conducted from nonreplacement random sampling of 6,000 tokens. An empirical *P* value was calculated by comparing the actual and the permutation results.

For images, statistical analysis was performed using 1-way ANOVA with Dunnett’s correction for multiple comparisons, with all disease samples compared only to control samples. A *P* value of less than 0.05 was considered statistically significant, and data represent mean ± SEM.

For comparison of inflammatory cell proportion, statistical analysis was performed using 1-way ANOVA with Dunnett’s correction for multiple comparisons. With all groups being compared only to normal skin samples. *P* less than 0.05 was considered statistically significant, and data are represented as mean ± SEM.

### Study approval.

DD, HHD, GD patients, and healthy adults provided written informed consent for skin biopsies. Tissues were anonymized for analysis and collected under IRB HUM00087890 at the University of Michigan.

## Author contributions

QRRC contributed to study conceptualization, methodology, formal analysis, investigation, writing the original manuscript draft, reviewing and editing the manuscript, and figure generation. HEB contributed to conceptualization, methodology, investigation, formal analysis, and reviewing and editing the manuscript. ZR contributed to methodology, data curation, and the investigation. JLK, XX, RMH, and ALP contributed to the investigation. LCT and JK contributed to data curation. PWH provided resources. LMG contributed to the investigation and reviewing and editing the manuscript. JEG contributed to study conceptualization, resources, reviewing and editing the manuscript, and funding acquisition. KJG contributed to study conceptualization, reviewing and editing the manuscript, and funding acquisition.

## Supplementary Material

Supplemental data

Supporting data values

## Figures and Tables

**Table 1:**
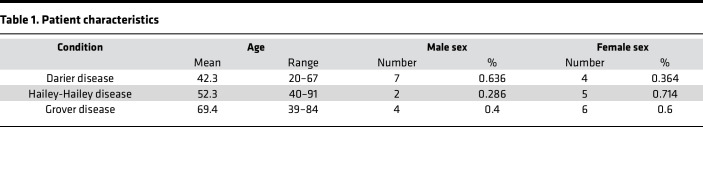
Patient characteristics

## Version 1 07/20/2023

In-Press Preview

## Version 2 08/22/2023

Electronic publication
