# Supplementary material for: Transcriptional profiling of rare acantholytic disorders suggests common mechanisms of pathogenesis
Source: JCI Insight. 2023 Aug 22;8(16):e168955. doi: 10.1172/jci.insight.168955 (PMC10543711; doi:10.1172/jci.insight.168955)
Supplement: Supplemental data [file jciinsight-8-168955-s192.pdf]

Supplemental Material

Transcriptional profiling of rare acantholytic disorders suggests common mechanisms of pathogenesis

Quinn R. Roth-Carter*, Hope E. Burks*, Ziyou Ren, Jennifer L. Koetsier, Lam C. Tsoi, Paul W. Harms, Xianying Xing, Joseph Kirma, Robert M. Harmon, Lisa M. Godsel, Abbey L. Perl, Johann E. Gudjonsson, Kathleen J. Green

*Authors contributed equally to this work.

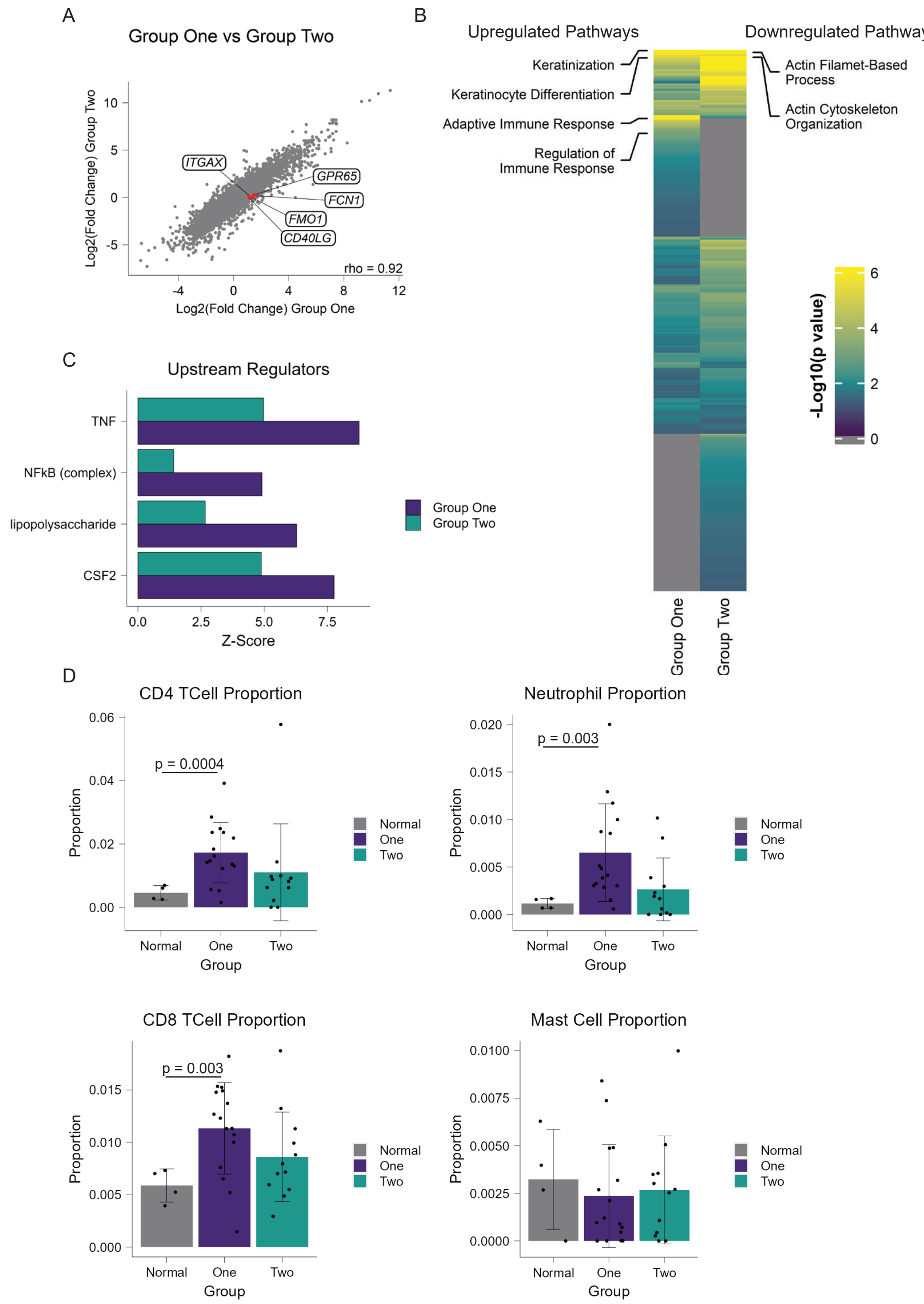

Supplemental Figure 1. DD, HHD and GD form two intermixed groups separated by level of inflammatory signatures. A) Correlation analysis of gene expression from group one and group two samples. Labeled genes are the top 5 differentially ranked genes from the spearman correlation, after filtering out lowly expressed genes and for genes that are significantly changed in at least one group. B) Heatmap showing GO BP pathways ranked by -Log$_{10}$(p value) for group one and group two. Selected upregulated pathways are noted left of the heatmap, while downregulated pathways are noted on the right. C) Z-score from IPA upstream regulator analysis, with a focus on inflammatory mediators. D) Predicted proportion of select inflammatory cells identified using CIBER-SORTx.

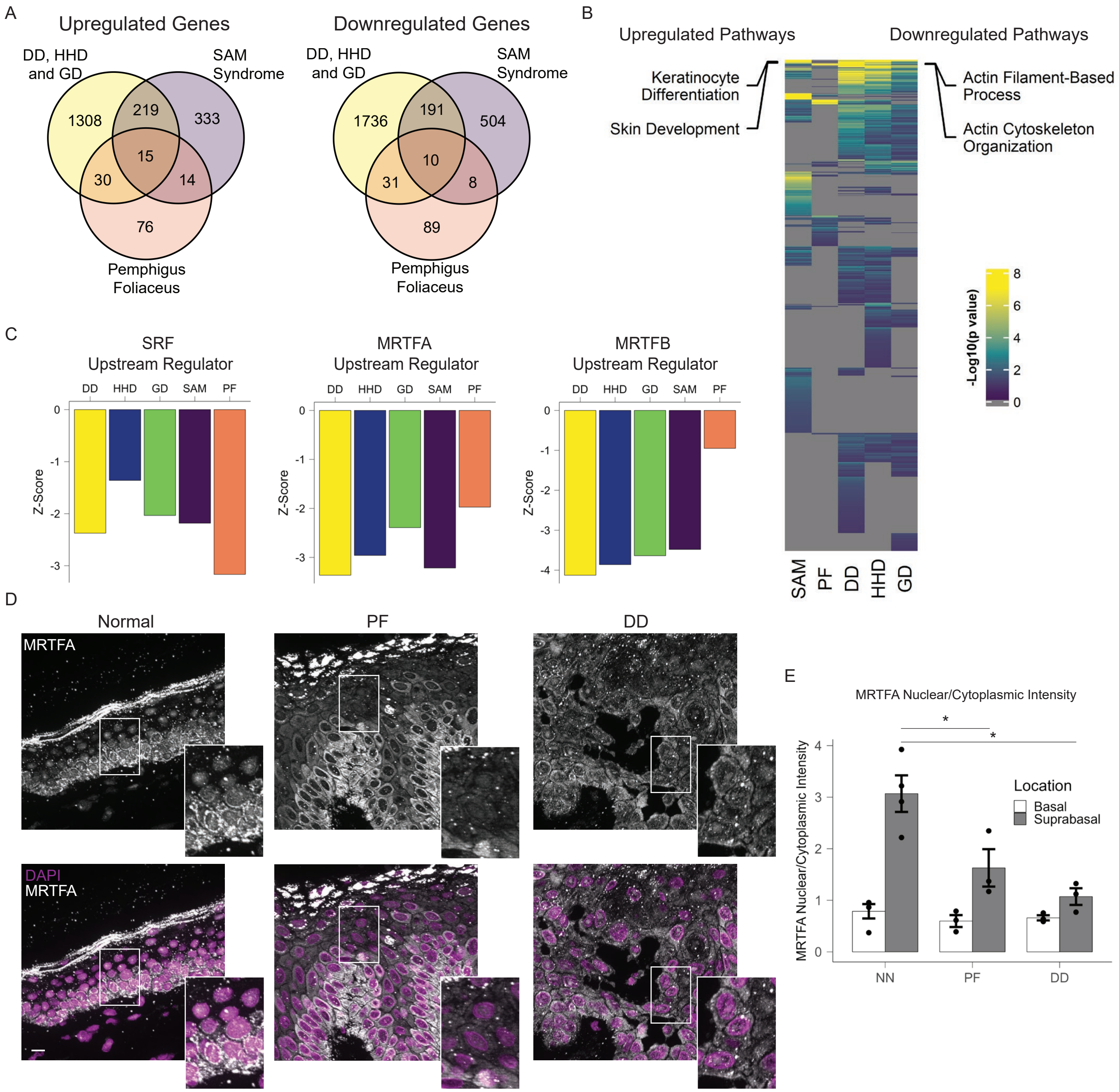

Supplemental Figure 2. Pemphigus foliaceus and SAM syndrome share changes in actin organization pathways with DD, HHD and GD. A) Venn diagram showing overlap in upregulated genes and downregulated genes in PF, SAM syndrome, DD, HHD, and GD samples. B) Heatmap showing GO BP pathways ranked by -$\log_{10}$(p value) for PF, SAM syndrome, DD, HHD and GD. Selected upregulated pathways are noted on the left of the heatmap, while downregulated pathways are noted on the right. C) IPA upstream regulator analysis showing z-score for SRF, MRTFA and MRTFB in PF, SAM syndrome, DD, HHD, and GD samples. D) Immunostaining for MRTFA in PF, DD, and NN skin. Quantification of MRTFA nuclear and cytoplasmic pixel intensities measured in basal and suprabasal cells. Scale bar = 20 μm.

Diagnostic Group	Age	Sex	Body Site
Grovers	72	f	Skin of right lower back, punch
Grovers	84	m	Skin of left abdomen, punch biopsy
Grovers	76	m	Skin of right flank, shave
Grovers	67	f	Skin of right upper arm, punch biopsy
Grovers	58	f	B. Skin of right mid submammary area − superior, wedge biopsy:
Grovers	77	m	Skin of left chest, punch
Grovers	82	f	Skin of mid back, punch biopsy
Grovers	57	f	Skin of right lower back, punch
Grovers	39	f	Skin of right arm, punch
Grovers	82	m	B. Skin of left middle back, shave
Hailey Hailey	40	m	B. Skin, right axilla, punch
Hailey Hailey	81	f	Skin, right back T12, punch
Hailey Hailey	45	f	Skin of left inframammary fold, punch
Hailey Hailey	47	f	Skin of left upper abdomen
Hailey Hailey	55	m	Skin of central chest, wide local excision
Hailey Hailey	54	f	Skin of left antecubital fossa, punch
Hailey Hailey	44	f	Skin of left inframammary region, punch
Dariers	24	m	Skin of right upper back, shave
Dariers	34	f	Skin of right back, punch
Dariers	49	m	Skin of right upper back, punch
Dariers	30	f	Skin of mid chest, punch
Dariers	67	m	Skin of central lower back, shave
Dariers	37	m	Skin of left shoulder, punch
Dariers	45	f	Skin of right upper abdomen, punch biopsy
Dariers	61	m	Skin of right forearm, shave
Dariers	49	m	Skin of left upper chest, shave
Dariers	49	m	Skin of left malar area 5.5 cm from ala, 7 cm from tragus, excision
Dariers	20	f	Trunk
NN	N/A	f	Flank
NN	N/A	f	Flank
NN	N/A	f	Flank
NN	N/A	f	Flank

Supplemental Table 1. Detailed characteristics of individual patient biopsy samples

Group	Mean Age	Female Sex		Male Sex		DD		HHD		GD	
		Number	%	Number	%	Number	% of DD	Number	% of HHD	Number	% of GD
Group 1	53.56	10	0.63	6	0.37	6	0.55	3	0.43	7	0.70
Group 2	55.67	5	0.42	7	0.58	5	0.45	4	0.57	3	0.30

Supplemental Table 2. Characteristics of samples between group 1 and group 2.
